# HIF-1α participates in secondary brain injury through regulating neuroinflammation

**DOI:** 10.1515/tnsci-2022-0272

**Published:** 2023-02-09

**Authors:** Xiaojian Xu, Mengshi Yang, Bin Zhang, Jinqian Dong, Yuan Zhuang, Qianqian Ge, Fei Niu, Baiyun Liu

**Affiliations:** Beijing Key Laboratory of Central Nervous System Injury, Beijing Neurosurgical Institute, Capital Medical University, Beijing, China; Beijing Key Laboratory of Central Nervous System Injury, Department of Neurosurgery, Beijing Neurosurgical Institute, Beijing Tiantan Hospital, Capital Medical University, Beijing, No. 119 South Fourth Ring West Road, Fengtai District, Beijing, 100070, China; Nerve Injury and Repair Center of Beijing Institute for Brain Disorders, Beijing, China; China National Clinical Research Center for Neurological Diseases, Beijing, China

**Keywords:** traumatic brain injury, HIF-1α, hypoxia, neuroinflammation, apoptosis, innate immune, 2-methoxyestradiol, secondary brain injury, GSEA, controlled cortical impact

## Abstract

A deeper understanding of the underlying biological mechanisms of secondary brain injury induced by traumatic brain injury (TBI) will greatly advance the development of effective treatments for patients with TBI. Hypoxia-inducible factor-1 alpha (HIF-1α) is a central regulator of cellular response to hypoxia. In addition, growing evidence shows that HIF-1α plays the important role in TBI-induced changes in biological processes; however, detailed functional mechanisms are not completely known. The aim of the present work was to further explore HIF-1α-mediated events after TBI. To this end, next-generation sequencing, coupled with cellular and molecular analysis, was adopted to interrogate vulnerable events in a rat controlled cortical impact model of TBI. The results demonstrated that TBI induced accumulation of HIF-1α at the peri-injury site at 24 h post-injury, which was associated with neuronal loss. Moreover, gene set enrichment analysis unveiled that neuroinflammation, especially an innate inflammatory response, was significantly evoked by TBI, which could be attenuated by the inhibition of HIF-1α. Furthermore, the inhibition of HIF-1α could mitigate the activation of microglia and astrocytes. Taken together, all these data implied that HIF-1α might contribute to secondary brain injury through regulating neuroinflammation.

## 1 Introduction

Traumatic brain injury (TBI) is the major cause of mortality and disability and poses an immense public health and economic burden to individuals and countries [1,2]. However, despite much effort, no effective medical treatment exists in large part due to the limited understanding of complex pathological processes after TBI [3,4]. TBI results from external mechanical force to the cerebral parenchyma followed by delayed secondary insults. Secondary brain injury deteriorates brain damage and causes progressive neuronal death and cognitive dysfunction [5]. Therefore, the protracted onset of secondary injury provides a promising window for reducing neurological deficits with TBI.

Hypoxia-inducible factor-1 (HIF-1) is the mast nuclear transcription factor, which orchestrates adaptive physiological and pathophysiological responses to hypoxia [6]. HIF-1 is composed of an oxygen-regulated alpha subunit (HIF-1α) and a constitutively expressed beta subunit (HIF-1β) [7]. Hypoxia-mediated post-translational modifications of HIF-1α are essential to maintain cellular and organismal homeostasis in response to internal and external stimuli [8]. It has been demonstrated that HIF-1 is involved in brain development [9], neurogenesis [10,11], and neuroprotection [12]. However, aberrant HIF-1 activation participates in multiple brain pathologies [13], including neurodegenerative diseases [14] and TBI [15].

Increasing evidence shows that HIF-1α is highly implicated in TBI [16]. However, the role of HIF-1α in TBI remains not to be completely understood. To further investigate HIF-1α-related biological processes after TBI, next-generation sequencing, coupled with cellular and molecular analysis, was utilized to interrogate vulnerable events in a rat controlled cortical impact (CCI) model of TBI treated with HIF-1α inhibitor 2-methoxyestradiol (2ME2). The CCI model is a well-established and commonly used animal model of TBI, and predominately produces a highly reproducible focal brain injury with better control over impact velocity, deformation depth, and dwell time [17,18]. The results revealed that HIF-1α inhibition could attenuate TBI-induced neuronal apoptosis, neuroinflammation, especially innate immunity responses, and glial activation, which indicated that targeting HIF-1α might be an alternative therapeutic strategy to treat TBI. Besides, it has been suggested that the complement system contributes to secondary brain injury [19]; however, its upstream regulatory pathways remain largely unknown. Our results suggested that HIF-1α could modulate the activity of the complement system.

## 2 Materials and methods

### 2.1 Animals

Adult male Sprague Dawley rats (8-week-old, 280–300 g) were provided by Beijing Vital River Experimental Animals Technology, Ltd, Beijing, China. All animals were housed under a controlled environment and allowed food and water ad libitum. Rats were randomly divided into three groups (*n* = 36 per group, total animal number *n* = 108): control, TBI, and 2ME2-treated. After the CCI procedure, 2ME2, dissolved in 10% dimethyl sulfoxide in Dulbecco’s phosphate-buffered saline (10 mg/kg, M6383-50 mg, sigma), was injected intraperitoneally.


**Ethical approval:** The research related to the use of animals has been complied with all the relevant national regulations and institutional policies for the care and use of animals. All the experimental processes were approved by the Beijing Neurosurgical Institute Animal Care and Use Committee (Approval No. 201802001) on June 6, 2018.

### 2.2 Controlled cortical impact model

Based on our previous experimental protocol, rats were anesthetized and maintained at a body temperature of 37.0 ± 0.5°C with a thermal pad [20]. The rat head was then fixed in a stereotaxic frame. After exposing the skull with a midline scalp, a craniotomy (diameter = 6 mm) was performed over the right parietal bone. Subsequently, CCI injury was induced with a PCI3000 PinPoint Precision Cortical Impactor (Hatteras Instruments, Cary, NC, USA) with the previous parameters (5 mm impactor tip diameter; 2 mm depth; 300 ms compression time; and 3 m/s velocity). Finally, the removed bone was replaced and fixed with wax. The rats of the control group underwent the same process without impact.

### 2.3 Tissue preparation

At 24 h post-injury, rats were anesthetized and perfused through the left cardiac ventricle with cold saline (0.9%). For RNA isolation, peri-injury cortices were collected and snap-frozen with liquid nitrogen immediately, and then stored at −80°C until RNA extraction. For immunofluorescence, mice were perfused with 0.9% cold saline and 4% paraformaldehyde (PFA) successively. Then, brains were post-fixed overnight in PFA. After 48-h cryoprotection in 30% sucrose at 4°C, 20 μm sections were acquired from brain tissues embedded in optimum cutting temperature compound.

### 2.4 Immunofluorescence

After heat-induced retrieval in sodium citrate buffer, slides were treated with blocking and permeabilization buffer (5% bovine serum albumin, 0.3% Triton X-100 in phosphate-buffered saline) at room temperature for 1 h. Then, the primary antibodies were added: mouse anti-HIF-1A (ab1), rabbit anti-glial fibrillary acidic protein (ab7260), rabbit anti-NeuN (ab177487), rabbit anti-Iba1 (Wako, 019-19741), rabbit anti-C3 (Proteintech, 21337-1-AP), and rabbit anti-Stat3 (Proteintech, 10253-2-AP) and incubated overnight at 4°C. Subsequently, the sections were incubated with secondary antibodies. Finally, 4’,6-diamidino-2-phenylindole (DAPI) was utilized to counterstain nuclei. For each primary antibody, three sections per brain were used. Images surrounding 1 mm^2^ from the margin of peri-contusion were taken with Nikon Instruments A1 confocal laser microscope (Nikon, Tokyo, Japan). Fluorescence intensity was quantified by using the ImageJ software (National Institutes of Health, NIH).

### 2.5 RNA isolation and quantitative real-time PCR (qRT-PCR)

RNA was extracted from rat brain cortex with Trizol reagent (Invitrogen) as described in our previous publication [21]. cDNA was synthesized using total RNA with the superscript II reverse transcription system according to the manufacturer’s instructions. Then, the thermal cycling protocol of qRT-PCR was performed as follows: 95°C for 15 s, 95°C for 5 s, and 60°C for 31 s, which was repeated for 40 cycles. The sequences of HIF-1α primers are listed as follows: forward primer: 5′-GCGGCGAGAACGAGAAGAAA-3′; reverse primer: 5′- TGTCAAGATCACCAGCACCT-3′. The expression of mRNA level was normalized with glyceraldehyde-3-phosphate dehydrogenase and determined by a comparative CT method.

### 2.6 Terminal deoxynucleotidyl transferase dUTP nick end labeling (TUNEL) assay

TUNEL staining was used to detect apoptosis in the peri-injury cortex by commercial TUNEL kit (In Situ Cell Death Detection Kit, Roche, Germany). Briefly, sections were subjected to permeabilization (0.1% Triton X-100, 0.1% sodium citrate) on ice. Sections were then treated with TUNEL reaction buffer in a humidified condition at 37°C for 60 min in the dark. Finally, nuclei were stained with DAPI. Images surrounding 1 mm^2^ from the margin of peri-contusion were taken with Nikon Instruments A1 confocal laser microscope (Nikon, Tokyo, Japan). The number of TUNEL-positive cells was measured using ImageJ software (National Institutes of Health, NIH).

### 2.7 RNAseq and analysis

RNAseq and functional analysis were performed as described in our previous work [3]. Briefly, the HISAT (2.0) package was used to align reads to the rat reference genome. The differential expression was finished with DESeq2 package (1.26.0). Gene set enrichment analysis was obtained with gene set enrichment analysis (GSEA) software (4.0.3). Significantly enriched gene sets were identified using false discovery rate <0.25.

### 2.8 Statistical analysis

R statistical software was used to perform statistical analysis. All data were presented as mean ± standard error of mean (SEM). For normal distribution data, the difference was determined by one-way analysis of variance (ANOVA) followed by the Tukey’s honestly significant difference (HSD) post hoc test, while nonparametric test was conducted with Kruskal–Wallis test followed by Steel–Dwass multiple comparison test. A *P*-value <0.05 was considered to be statistically significant.

## 3 Results

### 3.1 Increased accumulation of HIF-1α at the peri-injury site after TBI

**Figure 1:**
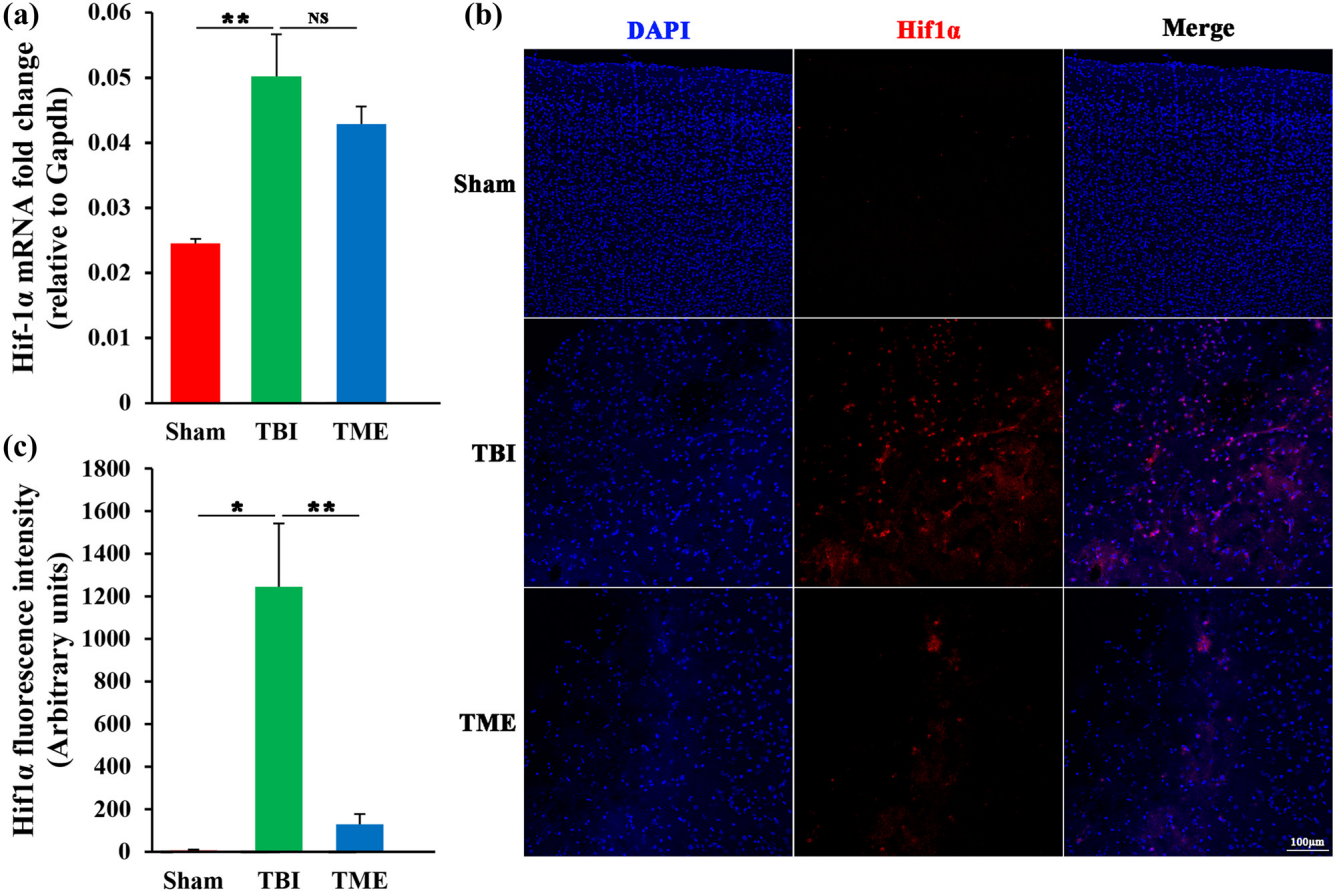
Increased accumulation of HIF-1α at the peri-injury site at 24 h post-injury. (a) qRT-PCR analysis of HIF-1α in Sham, TBI and 2ME2-treated rats (*n* = 3 rats per group). The difference was determined by one-way ANOVA (*F*(2,6) = 10.76, *P* = 0.0104) followed by the Tukey HSD post hoc test. (b) Representative photomicrographs of HIF-1α staining. (c) Quantification of HIF-1α fluorescence intensity (*n* = 4–9 rats per group). Significance was determined by nonparametric Kruskal–Wallis test (Kruskal–Wallis chi-squared = 17.221, df = 2, *P* = 0.0001822) followed by Steel–Dwass multiple comparison test. TBI, traumatic brain injury; 2ME2, 2-methoxyestradiol. Data are expressed as mean values ± SEM. *P < 0.05; **P < 0.01.

To determine the expression of HIF-1α after TBI, mRNA and protein levels of HIF-1α were measured using qRT-PCR and immunofluorescence, respectively. qRT-PCR results showed that mRNA levels of HIF-1α were significantly increased at 24 h post-injury (Figure 1a). 2ME2, an endogenous and naturally occurring metabolite of estradiol, can cross the blood–brain barrier (BBB) to exert their biological effects [22,23]. Although the bioavailability of 2ME2 is low (approximately 1.5%), efficient absorption of 2ME2 is observed in humans and rodent animals [24]. 2ME2 can reduce HIF-1α expression at the posttranscriptional level without affecting their transcription and stability through depolymerizing the microtubules, whereas there is no effect on HIF-1β levels and subcellular localization [25]. In the present work, 2ME2 could attenuate the mRNA expression of HIF-1α; however, the reduction did not reach statistical significance (Figure 1a). Consistently, the protein levels of HIF-1α were substantially upregulated after TBI and the expression was confined to peri-injury site (Figure 1b and c). Contrary to mRNA change, 2ME2 significantly reduced TBI-induced upregulation of HIF-1α (Figure 1b and c), which was in accordance with the post-translational effect of 2ME2 on HIF-1α. Collectively, TBI could increase HIF-1α expression, which was attenuated by HIF-1α inhibitor.

### 3.2 Inhibition of HIF-1α attenuated TBI-induced neuronal loss

**Figure 2:**
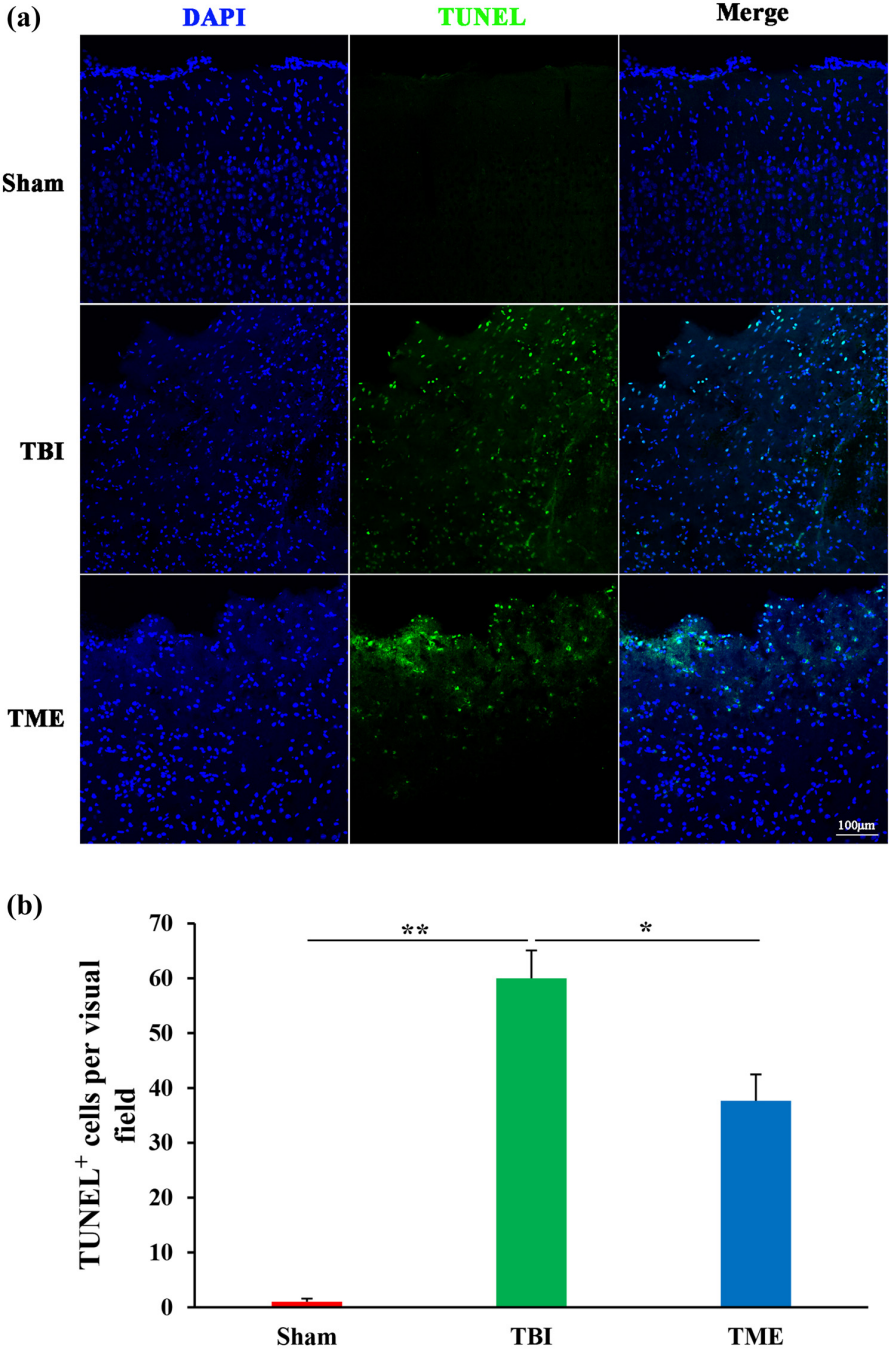
Inhibition of HIF-1α attenuated TBI-induced apoptosis. (a) Representative images of TUNEL staining. (b) Quantification of TUNEL-positive cells in Sham, TBI, and 2ME2-treated groups (*n* = 3–4 rats per group). The difference was determined by one-way ANOVA (*F*(2,7) = 46.49, *P* = 9.08 × 10^−5^) followed by the Tukey HSD post hoc test. TBI, traumatic brain injury; 2ME2, 2-methoxyestradiol. Data are expressed as mean values ± SEM. *P < 0.05; **P < 0.01.

**Figure 3:**
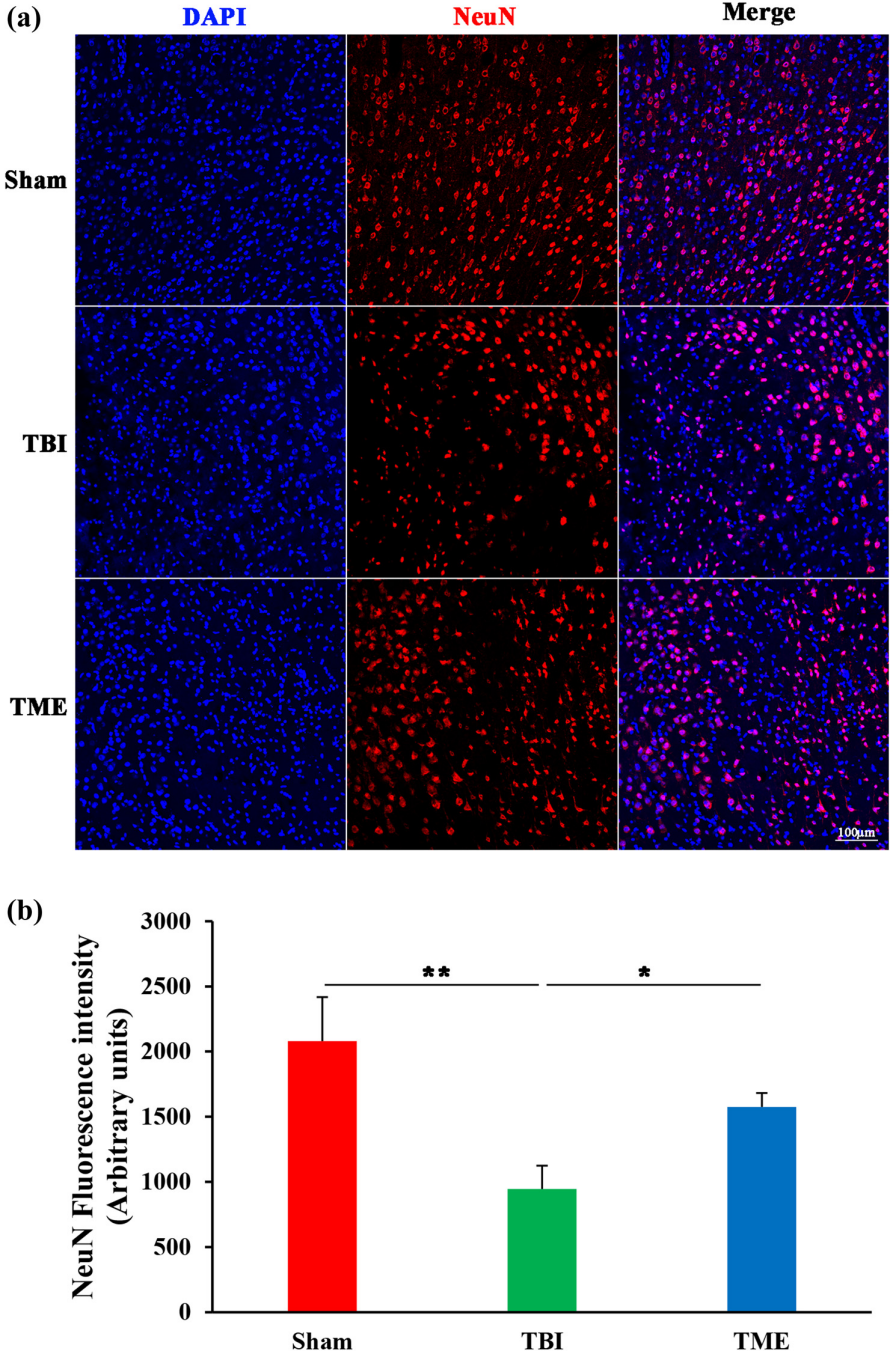
Inhibition of HIF-1α reduced TBI-induced neuronal loss. (a) Representative images of NeuN staining. (b) Quantification of NeuN fluorescence intensity (*n* = 5–6 rats per group). The difference was determined by one-way ANOVA (*F*(2,14) = 6.936, *P* = 0.00807) followed by the Tukey HSD post hoc test. TBI, traumatic brain injury; 2ME2, 2-methoxyestradiol. Data are expressed as mean values ± SEM. *P < 0.05; **P < 0.01.

Apoptosis largely accounts for TBI-induced cell death; therefore, the effect of HIF-1α on cell death was then investigated. TUNEL results demonstrated that TUNEL-positive cells at peri-injury site were significantly increased, which was dramatically mitigated by 2ME2 (Figure 2). Accordingly, it was observed that TBI caused massive neuron loss, which was also ameliorated by 2ME2 (Figure 3). These data suggested that HIF-1α mediated TBI-induced neuron loss.

### 3.3 HIF-1α was involved in neuroinflammation-related biological processes

**Figure 4:**
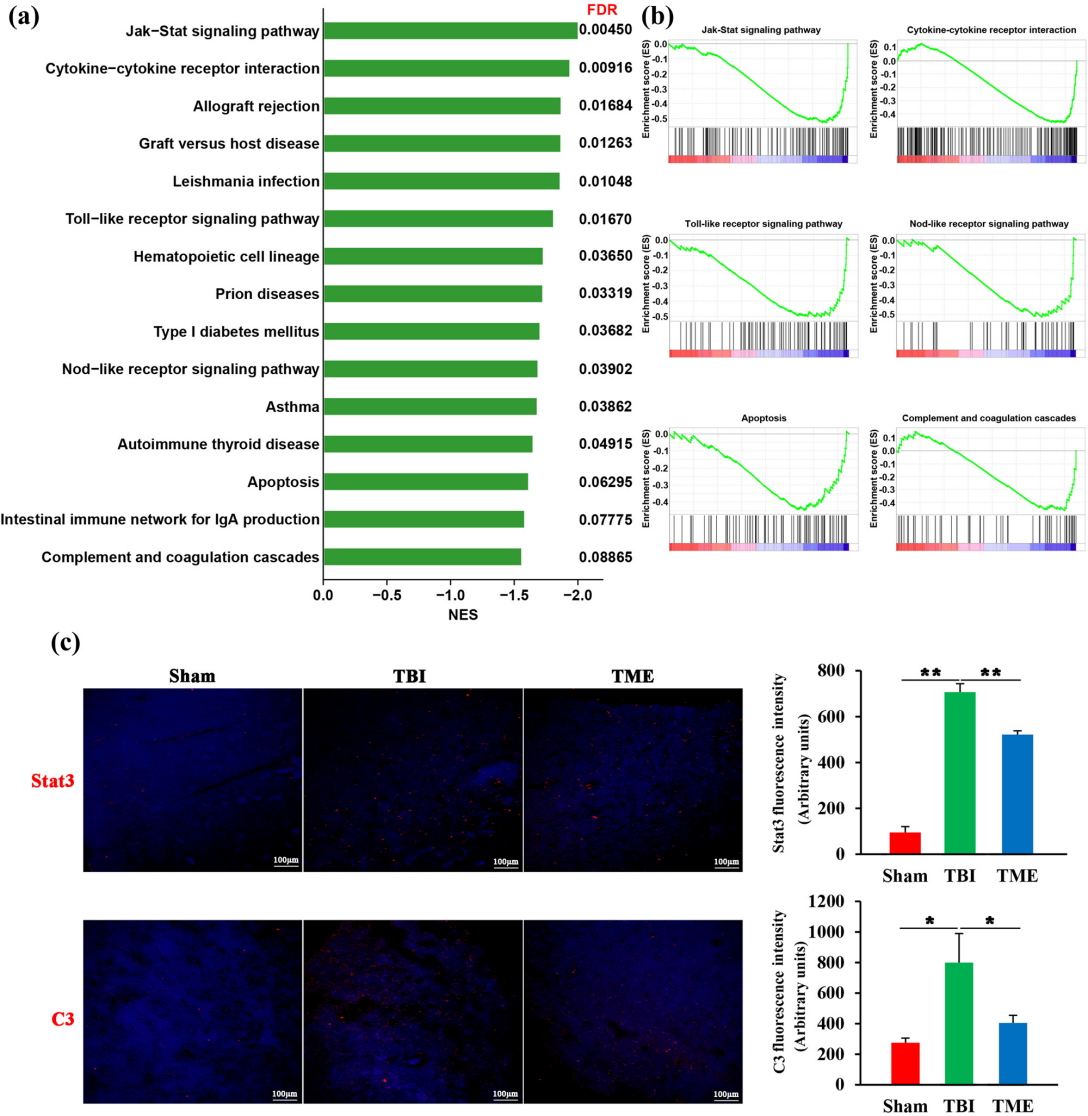
HIF-1α was involved in neuroinflammation-related biological processes. (a) Bar graph showing enriched kyoto encyclopedia of genes and genomes pathways obtained from GSEA. (b) Representative GSEA enrichment plots. (c) Representative images and quantification of Stat3 (*n* = 3 rats per group, *F*(2,6) = 148.2, *P* = 7.81 × 10^−6^) and C3 (*n* = 3–4 rats per group, *F*(2,8) = 7.655, *P* = 0.00139) staining. The difference was determined by one-way ANOVA followed by the Tukey HSD post hoc test. TBI, traumatic brain injury; 2ME2, 2-methoxyestradiol. Data are expressed as mean values ± SEM. *P < 0.05; **P < 0.01.

To deeply understand the underlying roles of HIF-1α in TBI, genome-wide transcriptome was adopted. In light of the powerful ability of GSEA in identifying the key events in health and disease states [26], GSEA was then used to annotate the biological processes after TBI. GSEA showed that inhibition of HIF-1α significantly attenuated innate immunity-related pathways activated by TBI, such as Jak-Stat signaling pathway, cytokine–cytokine receptor interaction, Toll-like and Nod-like receptor signaling pathway, and complement cascades (Figure 4a and b). Furthermore, the enrichment analysis was further confirmed by immunostaining of Stat3 and C3 (Figure 4c). Besides, apoptosis was also observed to be significantly inhibited by 2ME2, which was consistent with the aforementioned TUNEL results. Taken together, these data demonstrated that HIF-1α was involved in neuroinflammation-related biological processes.

### 3.4 HIF-1α-mediated activation of glial cells after TBI

**Figure 5:**
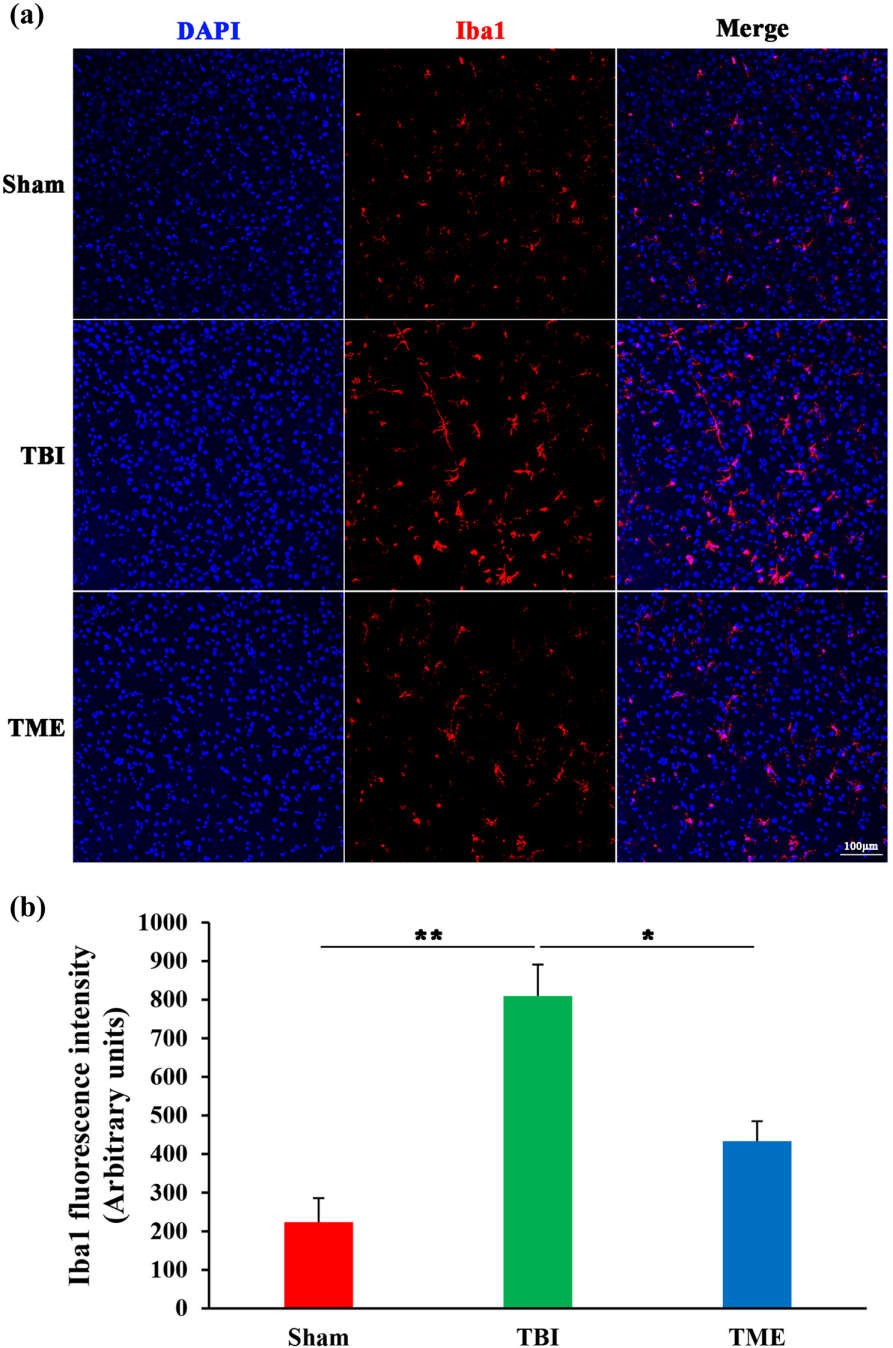
Inhibition of HIF-1α mitigated TBI-induced microglial activation. (a) Representative images of Iba1 staining. (b) Quantification of Iba1 fluorescence intensity (*n* = 3–4 rats per group). The difference was determined by one-way ANOVA (*F*(2,7) = 18.29, *P* = 0.00166) followed by the Tukey HSD post hoc test. TBI, traumatic brain injury; 2ME2, 2-methoxyestradiol. Data are expressed as mean values ± SEM. *P < 0.05; **P < 0.01.

**Figure 6:**
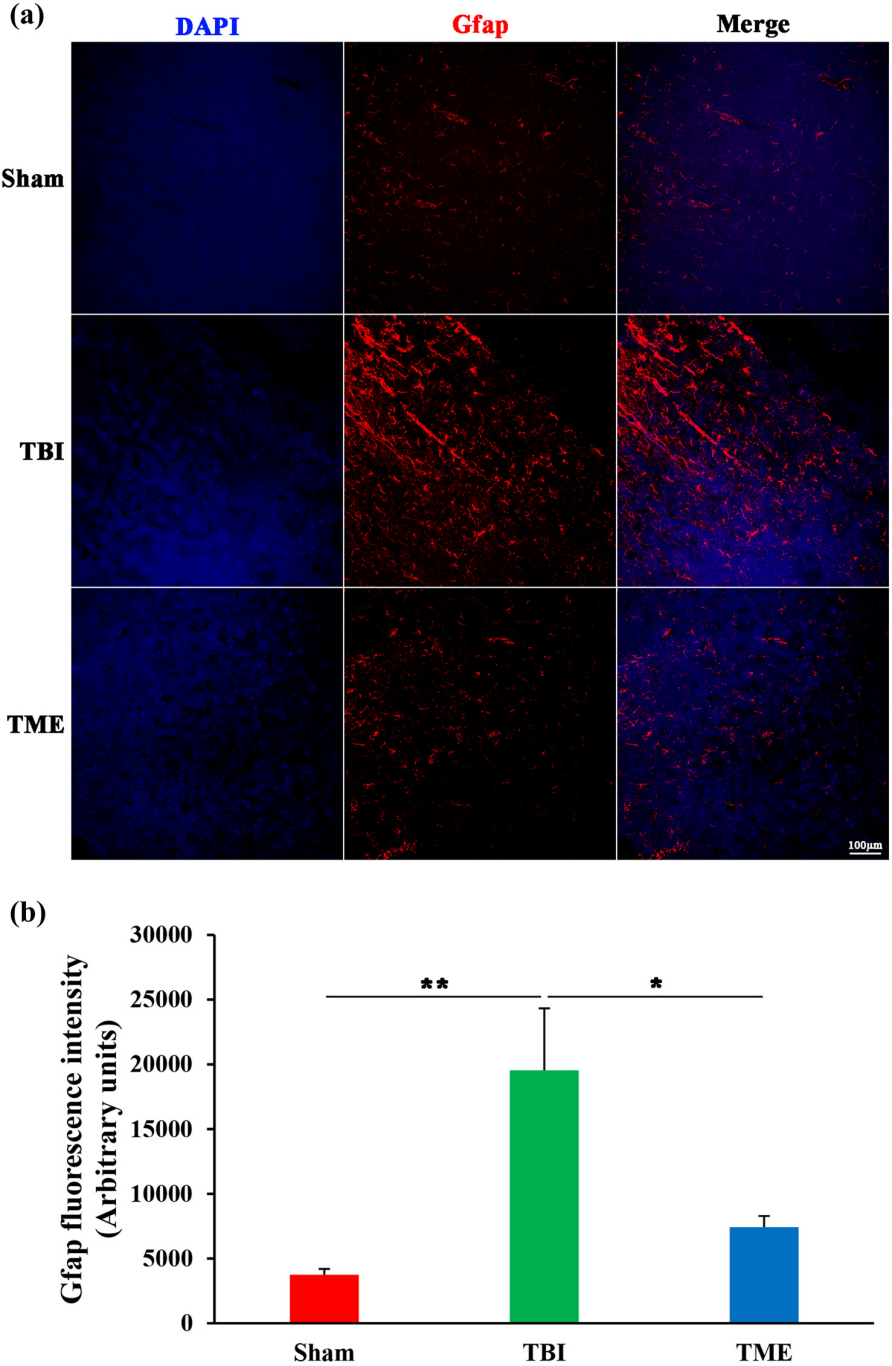
Inhibition of HIF-1α ameliorated TBI-induced activation of astrocytes. (a) Representative images of Iba1 staining. (b) Quantification of GFAP fluorescence intensity (*n* = 5–6 rats per group). The difference was determined by one-way ANOVA (*F*(2,13) = 6.997, *P* = 0.00866) followed by the Tukey HSD post hoc test. TBI, traumatic brain injury; 2ME2, 2-methoxyestradiol. Data are expressed as mean values ± SEM. *P < 0.05; **P < 0.01.

Glial cells, especially brain-resident microglia, are believed to be crucial players in evoking neuroinflammatory response after injury. Therefore, the effect of HIF-1α on glial cells, including astrocytes and microglia, was then evaluated. Iba1 immunostaining revealed that microglia were significantly activated following TBI. In contrast, the extent of microglia activation was enormously decreased by 2ME2 (Figure 5). The same phenomenon was also observed in astrocytes (Figure 6). Inhibition of HIF-1α after TBI could mitigate the activation of astrocytes. Collectively, these data implied that HIF-1α might promote neuroinflammation through glial cell activation.

## 4 Discussion

Increasing evidence shows that HIF-1α is implicated in multiple processes of TBI [27], which promoted us to further investigate the role of HIF-1α in TBI based on a rat model of TBI. The results showed that TBI increased the expression of HIF-1α at the peri-injury site at 24 h post-impact. Moreover, high expression of HIF-1α was associated with neuronal loss. In order to get more insight into the mechanism of HIF-1α-mediated brain damages, a combination of next-generation sequencing and HIF-1α inhibitor was adopted. Functional enrichment analysis demonstrated that HIF-1α could evoke innate inflammatory response at the acute phase of TBI, which might be attributed to glial activation after TBI. Collectively, the present work revealed that HIF-1α could participate in secondary brain injury through the induction of neuroinflammation.

TBI can result in cerebral hypoxia, which exerts a protective or detrimental effect on the clinical outcome of patients [16,27]. For severe TBI, approximately 70% of TBI patients suffered from cerebral hypoxia, and serum HIF-1α concentration highly correlated with poor outcome after head trauma [28]. HIF-1α could induce secondary brain injury through multiple processes, such as BBB disruption [29,30], brain edema [31,32], neuronal apoptosis [15,33,34,35], and neuroinflammation [36]. Brain edema is associated with high disability and morbidity, which is influenced by aquaporin expression and the integrity of BBB [37]. Following TBI, HIF-1α drove aquaporin 4 and 9 upregulation, thereby inducing cerebral edema and functional deficits. Besides, except for primary mechanical impact, the integrity of BBB was broken by an enhanced expression of matrix metalloproteinase-9 mediated by HIF-1α, which also accounted for brain edema. Additionally, HIF-1α could cause neuronal apoptosis by increasing the expression of pro-apoptotic BNIP3 and p53, and mediated tumor necrosis factor-related apoptosis-inducing ligand (TRAIL)-induced neuronal apoptosis through modulating TRAIL decoy receptor 1 (DcR1). More recently, Qian et al. found that HIF-1α promoted neuronal injury via enhancing vascular endothelial growth factor (VEGF) A expression, which was attenuated by MicroRNA-31.

Neuroinflammation is the important contributor of secondary brain injury. Bae et al. demonstrated that leucine-rich repeat kinase 2 (LRRK2) was the direct target of HIF-1α and transcriptionally activated in HIF-1α-dependent manner by TBI, which in turn promoted neuroinflammation and exacerbated brain damage [30]. Likewise, in our work, we found that the inhibition of HIF-1α ameliorated innate inflammatory response and attenuated microglia activation. Microglia, the principal resident immune cell type in the central nervous system, maintain the cerebral homeostasis by phagocytosis of apoptotic cells and debris [38]. Besides, microglia are also the key players in synaptic pruning during development and neuronal plasticity in adult brain as well as trophic neuronal support [39,40]. However, microglia abnormality is highly involved in multiple neurological disorders, including Alzheimer’s disease [41,42], Parkinson’s disease [43], and TBI-induced neurological dysfunction [44]. Interestingly, Yuan et al. unveiled that HIF-1α activated nod-like receptor protein-3 (NLRP3) inflammasome-mediated pyroptosis in microglia after TBI and promoted TBI-induced behavioral and cognitive deficits, which further enhanced our understanding of the detailed role of HIF-1α in secondary brain injury [36]. Besides, increasing evidence shows that the complement system plays a critical role in microglia-mediated TBI-related neurodegeneration [45]. On the one hand, microglia-derived C1q could induce neuron loss and chronic neuroinflammation, thereby leading to abnormal sleep spindles and epileptic spikes after TBI [46]; on the other hand, C3 opsonin triggered microglial phagocytosis of synapses and chronic cognitive deficits [47,48]. In the current work, we observed that upregulation of C3 protein could be attenuated by the inhibition of HIF-1α, which implied that HIF-1α might evoke neuroinflammation by modulating the complement system pathways. Taken together, these findings suggest that HIF-1α may modulate the TBI-induced neuroinflammation through influencing multiple innate immune pathways, such as NLRP3 inflammasome and complement system.

Besides, it has been demonstrated that HIF-1α might play the neuroprotective role in TBI. Treatment with HIF-1α activator or stability with post-translational modification such as S-nitrosylation could increase the expression of VEGF, erythropoietin, and phosphoinositide-dependent kinase-1 and 4 (PDK1 and PDK4), thereby providing neuroprotection after TBI [49,50]. Furthermore, reduced expression of HIF-1α-related neuroprotective molecules might be related to poor outcome in the TBI-afflicted elderly [51]. Intriguingly, HIF-1α also mediated solute carrier family 12 member 2 (Slc12a2)-induced hippocampal neurogenesis after TBI through stimulation of VEGF expression [11]. The divergence about the role of HIF-1α in TBI might be attributed to injury severity, timing of inhibition, impact methods, and species. Therefore, further investigation will be required to explore in-depth the exact role of HIF-1α in different phases of TBI and the detailed functions in different cell types of brain. Fortunately, the rapid development of single-cell sequencing will enhance the understanding of HIF-1α in TBI. Besides, increasing evidence shows that 17β-estradiol (E2) could exert protective effects on TBI through dampening BBB disruption, attenuating neuroinflammation, and oxidative stress [52]. Given that 2ME2 is an endogenous and naturally occurring metabolite of estradiol, the role of estrogen receptors (ERs) in HIF-1α-related neuroprotection should be investigated although 2ME2 has a low affinity for ERs and *in vivo* 2ME2 treatment decreases levels of ERs [53]. Exclusion of the potential of ERs influences with pharmacological and genetic approaches would highlight the role of HIF-1α in TBI.
